# Distribution characteristics of staghorn calculi bacteria and metabolic differences in patients of different genders

**DOI:** 10.1007/s00345-025-05569-6

**Published:** 2025-03-24

**Authors:** Zhibin Zheng, Weiguo Hu, Xijie Ding, Jian Li, Shaobo Zhou, Zhichao Chi, Wenjie Bai, Hongmei Jiang, Jianxing Li, Guojun Chen

**Affiliations:** 1 Department of Urology, Beijing Tsinghua Changgung Hospital, School of Clinical Medicine, Tsinghua University, Beijing, 102218 China; 2 Department of Urology, Qinghai University Affiliated Hospital, Xining, 810000 China

**Keywords:** Staghorn stones, Stone composition, Gender, Urease-producing bacteria

## Abstract

**Objective:**

The objective of this study was to examine the distribution patterns of bacteria and to elucidate the distinctions in stone composition and metabolism.

**Methods:**

A review of 205 cases of staghorn stones treated at Beijing Tsinghua Changgung Hospital from January 2016 to June 2024 was conducted. Data on preoperative mid-course urine culture, blood biochemistry, 24-hour urine and postoperative stone composition were collected. Stones with > 50% mixed stone components or single components were defined as main stone components. According to the main components, patients with staghorn calculi were divided into infectious and non-infectious stone groups, and the distribution of bacteria among different stone groups was analyzed. The metabolic differences between patients with staghorn calculi of different genders were analyzed according to the results of blood biochemistry and 24-hour urine metabolism.

**Results:**

The study population consisted of 88 males and 117 females with an average age of 53 ± 12 years. The most common components were magnesium ammonium phosphate hexahydrate and carbonated apatite (40%). The prevalence of calcium oxalate stones (16.6%) and uric acid stones (9.3%) was significantly higher in males (*P* < 0.05), while infectious stones (42.9%) were more prevalent in females (*P* < 0.05). The detection rate of *Proteus mirabilis* was more prevalent in the infectious stone group (26.2%), whereas the detection rate of *Ureaplasma urealyticum* (9%) and *Enterococcus faecalis* (6.9%) were more prevalent in non-infectious stone group (P*<0.05*). The levels of serum uric acid, serum creatinine, urinary calcium, urinary sodium, urinary phosphorus, urinary chloride and urinary uric acid were found to be significantly higher in males than in females (*P* < 0.05).

**Conclusion:**

The biochemical metabolism of patients with staghorn stones exhibits gender-specific differences, with a notable discrepancy in the distribution of bacteria. It is therefore necessary to refine the biochemical metabolic indicators and mid-stream urine culture tests.

## Introduction

Urinary stones represent a common clinical condition affecting the urinary system. The underlying aetiology involves complex interactions between multiple interrelated factors, including dietary patterns, lifestyle choices, genetic predisposition, and metabolic disorders. Global epidemiological data demonstrate a rising incidence of urinary stone disease, particularly associated with urbanization and lifestyle modifications [1, 2]. Clinically significant recurrence rates persist, with 50–70% of patients experiencing recurrence within 5–10 years post-diagnosis. Stone formation significantly impairs patients’ quality of life while creating substantial healthcare burdens. A 19-year UK retrospective cohort study demonstrated progressive increases in treatment costs, with total annual expenditure for stone management reaching £190–324 million in England (2010) [3].These findings underscore the critical need for improved preventive strategies and cost-effective management approaches.

Staghorn calculi represent a distinct category of urinary calculi characterized by their location within the renal pelvis, with branches extending into the renal calyces. These calculi are clinically classified into complete or partial subtypes [4]. Surgical management has long presented technical challenges in urological practice, as their complex morphology predicts both technical complexity and high postoperative complication rates. Staghorn calculi have a substantial stone burden and are susceptible to numerous complications as a consequence of suboptimal treatment. Associated complications include acute kidney injury (AKI) [5], while chronic inflammatory stimulation may induce malignant transformation in persistent cases. Percutaneous nephrolithotomy (PCNL) remains the first-line intervention for most staghorn calculi, though complex cases may require open surgical approaches [6].

Historically, infectious stones were considered the predominant type among staghorn calculi [5, 7]. However, emerging evidence indicates a marked increase in the proportion of metabolic stones. This epidemiological shift prompts a re-evaluation of whether the era of infectious stone predominance has transitioned into a new epidemiological phase. While existing studies have characterized staghorn calculi composition, gender-specific variations in component metabolism remain underexplored. Therefore, this study aims to investigate bacterial distribution patterns in staghorn calculi patients and examine gender-specific metabolic differences. These findings will establish a stronger theoretical foundation for optimizing therapeutic strategies and post-treatment recurrence prevention.

## Materials and methods

### Study patients

This study obtained ethical approval from the Research Ethics Committee of Tsinghua University Affiliated Beijing Tsinghua Changgung Hospital. We retrospectively analyzed 205 consecutive patients diagnosed with staghorn calculi at our institution from January 2016 to June 2024. Diagnosis was confirmed through computed tomography (CT), plain abdominal radiography (KUB), and renal ultrasonography. For patients with multiple clinical encounters, only data from the initial presentation were included in the analysis. All participants had complete baseline clinical profiles and no history of antibiotic use within 7 days prior to admission. Exclusion criteria comprised: (1) severe organ failure (cardiac, pulmonary, hepatic, or renal), (2) congenital renal disorders (medullary sponge kidney, renal tubular acidosis), (3) urological malignancies, (4) congenital urinary tract malformations.

### Mid-stream urine collection

Following hospital admission, 10 mL of midstream urine was aseptically collected from each participant using sterile collection tubes. Urine collection preceded antimicrobial administration, with samples transported for bacterial culture analysis. Samples underwent immediate processing within 1 h or refrigerated storage at 4 °C for delayed analysis within 8 h. Diagnostic thresholds for positive cultures were defined as > 10⁵ CFU/mL in female patients and > 10⁴ CFU/mL in male patients with complicated UTIs.

### Biochemistry of the blood and 24-hour urine collection

To ensure analytical validity, participants underwent a standardized 10-hour overnight fasting protocol before morning venous blood collection. Routine biochemical analyses included quantification of: serum calcium, creatinine, uric acid, parathyroid hormone (PTH), and estimated glomerular filtration rate (eGFR) assessment.

All 24-hour urine collections were initiated at 8 a.m. on the second day of the patient’s admission to the hospital and continued until 8 a.m. on the third day. Preservative-grade hydrochloric acid was added during the initial collection to stabilize urinary analytes. Urine composition analysis was conducted using standardized methodologies with certified automated analyzers. Quantified parameters included: total urine volume, calcium, phosphorus, sodium, potassium, chloride, and uric acid excretion rates. Participants received standardized instructions prohibiting vigorous physical activity and maintaining detailed dietary records throughout the collection period.

### Stones composition analysis

All patients underwent percutaneous nephrolithotomy (PCNL), and the removed stones were subsequently submitted for analysis. The stone composition was subjected to analysis using a Fourier transform infrared spectrometer (FTIR). In accordance with the European Urology guidelines [4], magnesium ammonium phosphate hexahydrate (MAP), carbonated apatite (CA), and ammonium acid urate (AAU) stones are classified as infectious stones. Calcium oxalate monohydrate (COM) and calcium oxalate dihydrate (COD) are classified as calcium oxalate stones. Stones with a main composition of more than 50% or a single component are defined as the main stone components.

### Statistical analyses

The statistical analysis was conducted using the SPSS 25.0 software. Measurement data was represented as mean ± standard deviation (SD), whereas counting data was represented as a percentage. Comparisons between groups were conducted using chi-squared tests, t-tests and Fisher’s exact test. In instances where data exhibited a non-normal distribution, this was indicated by [M (P25 ~ P75)]. Inter-group comparisons were conducted using the Mann-Whitney U test. The results were deemed statistically significant with a p-value of less than 0.05.

## Results

### Stones composition

The study cohort comprised 205 subjects (88 males, 117 females) demonstrating a 1:1.33 male-to-female (M: F ) ratio, with mean age 53 ± 12 years. The study revealed that 49 single-component staghorn stones (23.9%) were identified, with COM being the most prevalent (12.7%), followed by UA (4.9%). Furthermore, 156 mixed-component staghorn stones (76.1%) were identified. The most prevalent combination of two stone components was MAP and CA (40%), followed by COM and CA (12.7%). Among stones comprising three or more components, the most prevalent combination was COM, CA, and MAP, occurring in 7.8% of cases (Table 1).

**Table 1:** Component analysis of staghorn stones

Component					*N*		%
Single							
COM					26		12.7
UA					10		4.9
CYS					6		2.9
MAP					5		2.4
CA					2		1.0
Mixed							
MAP + CA					82		40.0
COM + CA					26		12.7
COM + UA					15		7.3
CA + COD					2		1.0
COM + COD					2		1.0
MAP + COM					1		0.5
CA + COM + MAP					16		7.8
CA + COM + COD					7		3.4
MAP + CA + AAU					5		2.4
Total					205		100

### The distribution characteristics of main components

Staghorn stones, whose main composition is MAP, CA, or AAU, were classified as infectious stones. The most frequently identified main component was infectious stones (59%), followed by calcium oxalate (26.3%) and uric acid (11.7%). Of the total number of infectious stones, 107 cases of MAP component (52.2%) and 14 cases of CA component (6.8%) were identified. The most prevalent main composition among male patients was calcium oxalate (16.6%), followed by infectious stones (16.1%), and uric acid (9.3%). In contrast, among female patients, the most prevalent stone component was infectious stones (42.9%), followed by calcium oxalate (9.8%) and uric acid (2.4%). Statistical analysis identified significant gender-specific disparities: calcium oxalate calculi (16.6% vs. 9.8%) and uric acid calculi (9.3% vs. 2.4%) demonstrated male predominance, whereas infectious stones demonstrated female predominance (42.9% vs. 16.1%, *P* < 0.05) (Table 2).

**Table 2:** The main components of Staghorn stones are distributed among different genders

Main component	Genders (%)			$\:{x}^{2}$ \Fisher	*P* value
	Male	Female	Total		
Infectious stones	33(16.1)	88(42.9)	121(59.0)	29.536	**< 0.001**
CaOx	34(16.6)	20(9.8)	54(26.3)	12.01	**0.001**
Uric acid	19(9.3)	5(2.4)	24(11.7)	14.57	**< 0.001**
Cystine	2(1.0)	4(2.0)	6(3.0)	-	0.702
Total	88(42.9)	117(57.1)	205(100)	-	-

### The distribution characteristics of bacteria

**Fig. 1:**
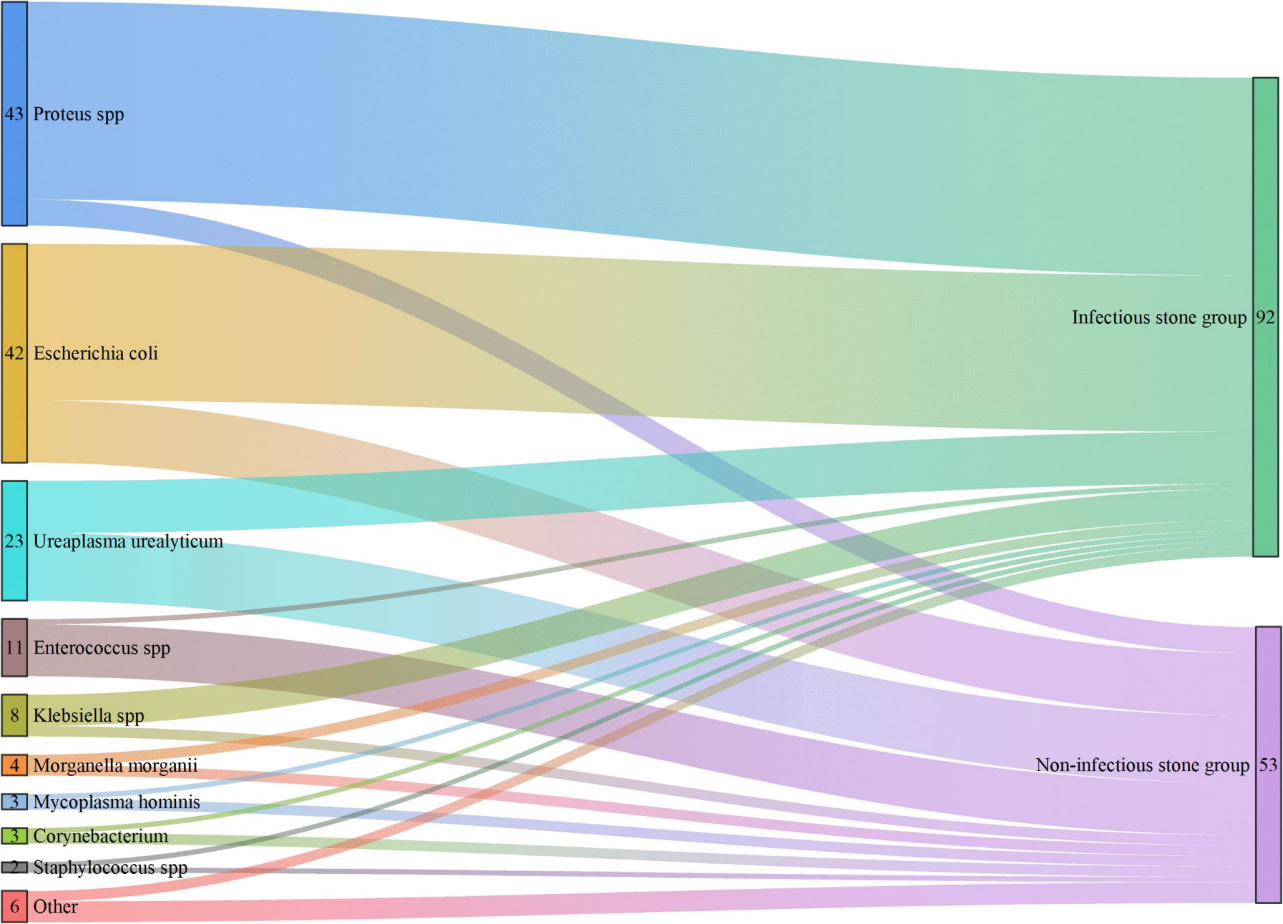
Distribution of microflora between infectious stone group and non-infectious stone group

A total of 145 patients with staghorn calculi had a positive mid-stream urine culture result. The most prevalent urease-producing bacteria were identified as *Proteus mirabilis* (29.6%, *n* = 43), followed by *Ureaplasma urealyticum* (15.9%, *n* = 23). The most prevalent non-urease-producing bacteria were *Escherichia coli* (29%, *n* = 42), followed by *Enterococcus faecalis* (7.6%, *n* = 11). Staghorn stones, whose primary composition is MAP, CA, or AAU, were classified as an infectious stone group, while the remainder were classified as a non-infectious stone group. The detection rate of *Proteus mirabilis* was significantly higher in the infectious stone group (26.2% vs. 3.4%), while the detection rate of *Ureaplasma urealyticum* (9% vs. 6.9%) and *Enterococcus faecalis* (6.9% vs. 0.7%) was significantly higher in the non-infectious stone group (*P* *< 0.05*) (Table 3; Fig. 1).

**Table 3:** Distribution of microflora between infectious stone group and non-infectious stone group

Bacteria	Stone composition grouping (%)			$\:{x}^{2}$\Fisher	*P* value
	Infectious stone group	Non-infectious stone group	Total		
Proteus spp	38(26.2)	5(3.4)	43(29.6)	16.37	**< 0.001**
Escherichia coli	30(20.7)	12(8.3)	42(29.0)	1.62	0.203
Ureaplasma urealyticum	10(6.9)	13(9.0)	23(15.9)	4.70	**0.030**
Enterococcus spp	1(0.7)	10(6.9)	11(7.6)	-	**< 0.001**
Klebsiella spp	6(4.1)	2(1.4)	8(5.5)	-	0.711
Morganella morganii	2(1.4)	2(1.4)	4(2.8)	-	-
Mycoplasma hominis	1(0.7)	2(1.4)	3(2.1)	-	-
Corynebacterium	1(0.7)	2(1.4)	3(2.1)	-	-
Staphylococcus spp	1(0.7)	1(0.7)	2(1.4)	-	-
Other*	2(1.3)	4(2.7)	6(4.0)	-	-
Total	92(63.4)	53(36.6)	145(100)	-	-

* Other bacteria include Pseudomonas aeruginosa, Burkholderia gladioli, and Fungi

### A comparative metabolic analysis of males and females

The results of the T-tests and Mann-Whitney U tests indicated that the uric acid and serum creatinine levels of male patients were significantly higher than those of female patients in terms of blood biochemistry (*P* *< 0.05*). The levels of urinary calcium, sodium, phosphorus, chlorine, and uric acid in males from 24-hour urine collections were significantly higher than in females (*P* *< 0.05*). However, there were no statistically significant differences in hypertension, diabetes, obesity, PTH, serum calcium, eGFR, urinary potassium, and 24-hour urine volume between genders (*P* *> 0.05*) (Table 4).

**Table 4:** Metabolic characteristics in patients with Staghorn calculi of different genders

Metabolic index	Genders		*P* value
	Male(*n* = 88)	Female (*n* = 117)	
Diabetes			0.448
Yes	13(6.4)	22(10.7)	
No	75(36.6)	95(46.3)	
Hypertension			0.214
Yes	32(15.6)	33(16.1)	
No	56(27.3)	84(41.0)	
Obesity (≥ 30 kg/m^2^)			0.294
Yes	6(2.9)	13(6.4)	
No	82(40.0)	104(50.7)	
Serum			
Creatinine (µmol/L)	126.33 ± 102.61	98.38 ± 93.01	**0.043**
eGFR(mL/min)	75.85 ± 32.72	77.65 ± 31.71	0.692
Uric acid (µmol/L)	405.18 ± 105.54	354.27 ± 106.47	**0.001**
Calcium (mmol/L)	2.26 ± 0.12	2.25 ± 0.13	0.677
PTH (ng/L)	46.4(31.98,67.88)	46.75(35.93,64.08)	0.536
24-hour urine			
Calcium (mmol /d)	3.69 ± 2.36	2.97 ± 1.93	**0.021**
Sodium (mmol/d)	176.06 ± 76.24	150.91 ± 67.68	**0.014**
Potassium (mmol/d)	31.91 ± 17.88	29.88 ± 12.26	0.336
Phosphorus (mmol /d)	17.34 ± 6.05	13.8 ± 5.97	**< 0.001**
Chloride (mmol/d)	146.18 ± 67.51	124.7 ± 56.44	**0.014**
Uric acid (µmol /d)	2922.35 ± 1070.73	2585.19 ± 973.3	**0.02**
Urine volume (ml)	2071.48 ± 740.15	2237.18 ± 476.03	0.116

## Discussion

The global burden of urolithiasis continues to escalate, paralleling socioeconomic development and modernization of dietary patterns. On average, one in 11 people in the United States experiences the pain of urolithiasis [8, 9]. Nationwide epidemiological surveys in China reveal a comparable renal calculi prevalence of 11.4% [10]. This escalating disease burden imposes substantial socioeconomic pressures on healthcare systems worldwide. Staghorn calculi management remains a formidable clinical challenge due to their complex spatial architecture. A systematic characterization of their metabolic profiles could inform evidence-based clinical protocols for therapeutic intervention and recurrence mitigation. Our findings indicate that staghorn stones remain the most prevalent form of infectious stones. Additionally, biochemical metabolic differences were observed among patients of varying genders, and the bacterial distribution of staghorn stones exhibited considerable heterogeneity.

We found that infectious stones were more common in staghorn stones. Emerging epidemiological evidence suggests metabolic stones prevalence may escalate with socioeconomic development and dietary patterns [11]. However, it is clear that infected stones remain the most common type of staghorn stones. A recent retrospective cohort study also demonstrated that struvite stones are more prone to developing staghorn stones than matrix stones or calcareous stones (56.5% vs. 21.7% vs. 18.8%, *P* < 0.05) [7]. Infectious stones demonstrate accelerated progression kinetics, achieving radiologically detectable volumes within 4-week intervals. This rapid progression window underscores the clinical urgency for early intervention. Staghorn calculi predominantly containing calcium oxalate monohydrate (COM) demonstrate clinical predominance over calcium oxalate dihydrate (COD) variants. This divergence originates from distinct pathophysiological mechanisms governing their crystallization pathways. COM crystallization principally correlates with diminished urinary inhibitors (e.g., citrate, magnesium), whereas COD pathogenesis is driven by hypercalciuric states. Crystallographic analysis reveals COM’s structural stability versus COD’s metastable configuration. These thermodynamic gradients facilitate phase transformation from COD to COM within dynamic urinary microenvironments [12, 13].

This study revealed a significantly higher incidence of calcium oxalate in male patients compared to females. These findings are consistent with those of Xie et al. [2], who demonstrated that a higher proportion of calcium oxalate stones were present in males (69.0%) compared to females (57.3%) (*P* *= 0.001*). This may be closely related to lifestyle and dietary habits, such as a diet high in sodium, which has been demonstrated to result in a reduction in calcium reabsorption in the proximal tubule. This can result in an increased excretion of urinary calcium and sodium [14]. Additionally, a high intake of animal protein has been demonstrated to elevate the excretion of urinary calcium. Therefore, controlling the intake of sodium salt and limiting the amount of animal protein can effectively prevent the formation of calcium oxalate stones [15]. Additionally, we observed that the urinary sodium and calcium levels in male patients were markedly elevated in comparison to those in female patients. This finding may provide a potential explanation for the elevated incidence of calcium oxalate staghorn calculi in male patients [16]. Moreover, it has been postulated that the influence of sex hormones may result in variations in stone formation [17]. The presence of androgens has the capacity to elevate serum levels of oxalic acid in a manner that results in augmented oxalic acid excretion. In contrast, estrogen may increase the excretion of urinary inhibitors, thereby inhibiting the formation of calcium oxalate stones. The study revealed that blood uric acid and urinary uric acid levels were significantly higher in male patients than in female patients. This finding may also explain the high prevalence of uric acid stones in male patients. A study by Wen et al. [18] indicated that individuals with elevated uric acid levels are at an increased risk of developing uric acid stones. The consumption of foods of animal origin and legumes both contribute to the development of hyperuricaemia. Consequently, dietary management represents a valuable strategy for the prevention of uric acid stone formation [19]. The study found that metabolic markers were higher in male participants. These findings provide a mechanistic basis for the proposition that dysmetabolism may promote the progression of staghorn calculi through both independent or synergistic pathways [20]. This also lends further support to the “metabolism-infection synergy hypothesis.” Abnormal metabolism can be a contributing factor to the development of stones; and it may also create an alkaline environment conducive to infection, thus leading to a vicious cycle of “metabolism-infection.” This suggests that metabolic interventions may still be necessary to prevent recurrence, even in cases where infectious stones are predominant.

Gender-stratified analysis revealed 2.7-fold higher prevalence of infectious calculi in females versus males (42.9% vs. 16.1%, *p* < 0.001). This discrepancy may be attributable to chronic urinary tract infections [21]. The female urethral anatomy presents specific characteristics that make bacterial infections more likely to develop. This can result in the formation of infectious stones [22]. By analysing the distribution characteristics of bacteria in infectious and non-infectious stones, it was found that *Proteus mirabilis* was the most prevalent urease-producing bacteria, which was consistent with the findings of previous studies in the field [23, 24]. Accordingly, for patients presenting with staghorn stones and urinary tract infections, an appropriate choice of antibiotics, made on the basis of the results of a bacterial etiology investigation of the urinary tract, can reduce the incidence of perioperative infections and stone recurrence rates [25].The development of infectious stones is frequently associated with the presence of urease-producing bacteria [26]. However, our findings indicate that *Ureaplasma urealyticum* was more prevalent in the non-infectious stone groups, we had to consider the reasons for the unusually high detection rate of *Ureaplasma urealyticum* in the non-infectious stone group, suggesting that this bacterium may be involved in stone formation through non-urease-dependent pathways. A recently conducted study demonstrated that metabolic abnormalities are prevalent in patients with mixed stones, comprising both infected and metabolic components, as well as in patients with purely metabolic stones [20].These findings suggest that individualized therapy should combine metabolic assessment with bacteriological findings. Additionally, *Enterococcus faecalis* was observed to be more prevalent in the non-infectious stone group, which suggests a potential need for therapeutic strategies that target Gram-positive bacteria in patients with such renal stones in clinical settings. Molecular diagnostics or stone microbiome analysis may provide more accurate bacterial information; however, the selection of mid-stream urine cultures was primarily informed by considerations of clinical maneuverability and cost-effectiveness. Mid-stream urine culture is currently the preferred diagnostic method for infections in primary hospitals in China, and the results of this study may directly inform the initial choice of antibiotics. Subsequent studies will integrate stone microbiome analysis (e.g., 16 S rRNA sequencing) and direct stone microbiome culture comparisons to comprehensively assess bacterial distribution.

The 24-hour urine metabolism test is a valuable diagnostic instrument that can provide insights into the metabolic state and directly reflect environmental factors that may contribute to the formation and growth of stones. Accordingly, regulating the urine composition in accordance with the metabolic outcomes can prove an efficacious method of preventing the development of urinary stones [27]. We found that the levels of urinary calcium, sodium, potassium, phosphorus, chloride, and uric acid were significantly higher in males than in females (*P* < 0.05). This suggests that dietary metabolism may be a factor that should not be overlooked. An elevated urinary calcium excretion in male patients may precipitate an increased propensity to develop calcium oxalate stones [28]. Similarly, an augmented urinary uric acid excretion in male patients may also be linked to an augmented likelihood of forming uric acid stones. It can be reasonably deduced that modifying unhealthy dietary practices and enhancing water consumption can effectively prevent the supersaturation of crystals, thereby reducing the likelihood of stone formation [29, 30].

This study is a retrospective case analysis conducted at a single center, which may lead to selective bias in the results. This may prevent the study from reflecting the stone formation in different regions and among different races. Methodological constraints precluded 24-hour urinary oxalate and citrate excretion analysis, compromising comprehensive metabolic pathway characterization. Furthermore, the failure to account for chronic pharmacotherapy (e.g., loop diuretics, uric acid lowering drugs) introduces potential confounding effects on crystalloid composition profiles. These limitations underscore the need for prospective multicenter designs with standardized metabolic workups and medication exposure documentation. Notwithstanding these constraints, our investigation provides valuable insights into gender-specific pathophysiological mechanisms and microbiome-stone interactions in East Asian populations. These findings establish critical framework for developing ethnicity-tailored prevention protocols and precision therapeutic strategies.

## Conclusion

In light of the observed gender-related disparities, metabolic abnormalities manifested more distinctly in male patients. Consequently, meticulous dietary management and uric acid monitoring is advised for male patients. Conversely, due to the high percentage of infected stones in women, it is recommended that female patients be screened for urinary tract infections and receive antibiotic prophylaxis.

## Data Availability

The data used or analyzed in this study are available from the corresponding author upon reasonable request.

## Abbreviations

CaOx: Calcium oxalate
COD: Calcium oxalate dihydrate
COM: Calcium oxalate monohydrate
UA: Uric acid
MAP: Magnesium ammonium phosphate hexahydrate
AAU: Ammonium acid urate
CYS: Cystine
CA: Carbonate apatite
PCNL: Percutaneous nephrolithotomy
FTIR: Fourier-transform infrared
eGFR: estimated glomerular filtration rate
PTH: Parathyroid hormone
